# Iron Deprivation in Cancer––Potential Therapeutic Implications

**DOI:** 10.3390/nu5082836

**Published:** 2013-07-24

**Authors:** Jessica L. Heath, Joshua M. Weiss, Catherine P. Lavau, Daniel S. Wechsler

**Affiliations:** 1 Division of Pediatric Hematology-Oncology, Department of Pediatrics, Duke University Medical Center, Durham, NC 27710, USA; E-Mails: jessica.heath@dm.duke.edu (J.L.H.); jmweiss18@me.com (J.M.W.); catherine.lavau@duke.edu (C.P.L.); 2 Department of Pharmacology & Cancer Biology, Duke University Medical Center, Durham, NC 27710, USA

**Keywords:** iron, chelation, cancer, leukemia, neuroblastoma, ribonucleotide reductase

## Abstract

Iron is essential for normal cellular function. It participates in a wide variety of cellular processes, including cellular respiration, DNA synthesis, and macromolecule biosynthesis. Iron is required for cell growth and proliferation, and changes in intracellular iron availability can have significant effects on cell cycle regulation, cellular metabolism, and cell division. Perhaps not surprisingly then, neoplastic cells have been found to have higher iron requirements than normal, non-malignant cells. Iron depletion through chelation has been explored as a possible therapeutic intervention in a variety of cancers. Here, we will review iron homeostasis in non-malignant and malignant cells, the widespread effects of iron depletion on the cell, the various iron chelators that have been explored in the treatment of cancer, and the tumor types that have been most commonly studied in the context of iron chelation.

## 1. Introduction

Iron is an essential element for normal cellular function. It exists biologically in two forms: ferrous (2+) and ferric (3+) iron. The ability of iron to change between these two states allows it to participate in a wide variety of cellular processes. Iron is a cofactor for several key enzymes in cellular respiration and metabolism, including enzymes of the citric acid cycle, and also ribonucleotide reductase. This latter enzyme catalyzes the reduction of ribonucleotides to deoxyribonucleotides, which is the rate-limiting step in DNA synthesis. Iron is also required for macromolecule biosynthesis, necessary for cell growth and division.

Because of their rapid cell growth and proliferation, neoplastic cells display an increased requirement for iron. To reflect this increased requirement, iron homeostasis is altered in neoplastic cells in a number of ways, including upregulation of surface transferrin receptor. Since iron plays a critical role in processes involved in cell growth and proliferation, and because of the enhanced dependence on iron metabolism in neoplastic cells, there has been significant interest in the possibility that iron depletion strategies might have an anti-proliferative effect on tumor cells. After reviewing iron hemostasis in normal and neoplastic cells, we will discuss the role of iron depletion as a potential therapeutic intervention in a variety of cancer types.

## 2. Normal Iron Homeostasis

Iron homeostasis is a tightly regulated process. Both iron excess and deficiency are toxic to individual cells and to the host. Because humans do not have an efficient mechanism by which to excrete excess iron, the primary method of regulating total body iron stores is via modulation of iron absorption through the duodenum. Iron may be present in multiple forms within the intestinal lumen. Heme iron is derived from hemoglobin and myoglobin, and is primarily found in meat. Non-heme iron includes ferrous and ferric iron, and may be derived from plant or animal products.

The absorption of heme iron remains poorly understood. It is likely that heme iron is taken up by enterocytes via receptor-mediated endocytosis [1]. While two receptors capable of binding heme have been identified at the apical surface of enterocytes, these are not high affinity receptors and are not likely to be the primary route by which heme iron is absorbed. Once heme-associated iron enters the enterocyte, it is liberated by heme oxygenase. It then enters the labile intracellular iron pool to be either stored within ferritin or exported from the cell as described below.

**Figure 1:**
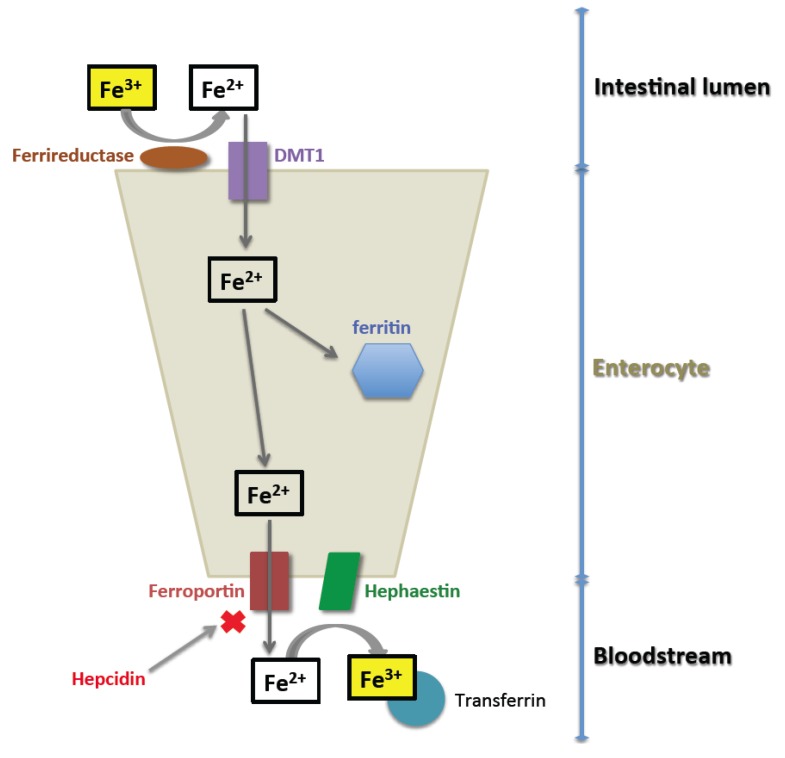
Intestinal absorption of iron. Ferric (Fe^3+^) iron present in the intestinal lumen is reduced to ferrous (Fe^2+^) iron, which can be transported by DMT1 across the apical surface of the enterocyte. Intracellular iron may be stored in ferritin, or transported out of the cell via ferroportin. After its release from the cell, it is oxidized by hephaestin and bound to transferrin for transport through the body.

The mechanisms of intestinal absorption of non-heme iron are better understood (Figure 1). Non-heme iron is absorbed through the intestinal epithelium via a tightly regulated, transporter-mediated mechanism. Most dietary non-heme iron is present in the intestinal tract as ferric iron (Fe^3+^) [1]. The apical surface of duodenal enterocytes contains DMT1 transporters, which act in concert with a ferrireductase to import ferrous iron (Fe^2+^) into the cell. From here, iron may be stored within the enterocyte complexed as ferritin, or transported across the basolateral membrane via ferroportin. At the basolateral membrane, hephaestin, a transmembrane copper-dependent ferroxidase homologous to ceruloplasmin, converts ferrous iron (Fe^2+^) to ferric (Fe^3+^) iron, which can be taken up by transferrin in the blood. Any iron that is not absorbed across the basolateral membrane is lost with the periodic sloughing of the intestinal epithelium.

The export of iron via ferroportin is primarily regulated by hepcidin [2]. Hepcidin is produced by hepatocytes in response to elevated iron levels, and its presence results in internalization and degradation of ferroportin. This occurs in both the enterocyte and the macrophage, which is responsible for scavenging and then recycling iron from senescent erythrocytes. The result of increased hepcidin production is therefore a decrease in serum iron and ultimately a decrease in total body iron.

**Figure 2:**
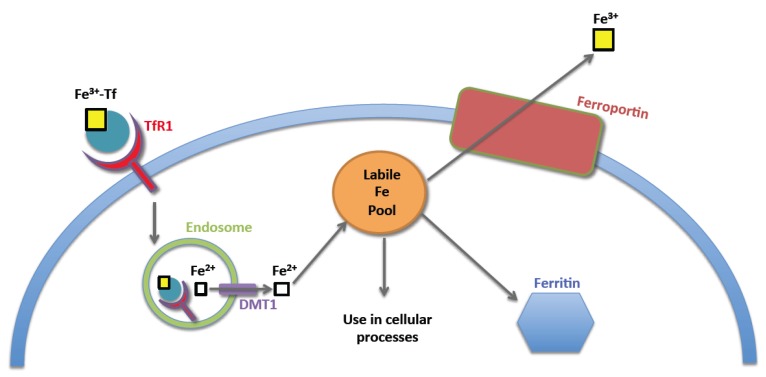
Cellular iron metabolism. Iron laden transferrin is taken up by the cell via clathrin-mediated endocytosis of transferrin receptor-1 (TfR1). Acidification of the resultant endosome releases iron from TfR. Iron is transported out of the endosome via DMT1, and becomes part of the labile iron pool. Iron may then be used in a variety of cellular processes, stored as ferritin, or exported out of the cell via ferroportin.

Once iron is exported from the enterocyte through ferroportin, it is transferred to transferrin (Tf) and is able to safely pass through the circulation in this bound state. The transferrin protein is 679 amino acids in length, and is able to effectively bind and transport two molecules of ferric iron [3]. Diferric Tf can bind to either of two cell surface transferrin receptors, TfR1 or TfR2, and is internalized. Clathrin-mediated endocytosis of the Tf-TfR1 complex is the primary mechanism by which cells are able to take up iron. Once internalized, iron dissociates from Tf as a result of acidification of the endosome and is released into the cytosol through DMT1 [4]. Apo-Tf-TfR1 (*i.e.*, TfR1 bound to Tf that is no longer bound to iron) is then recycled to the cell surface. Once in the cytosol, iron becomes part of the *labile iron pool*, a relatively poorly characterized source of free iron within the cytosol. The presence of large quantities of free iron in the cell is toxic, because of iron’s ability to participate in free radical formation via Fenton chemistry. The oxidation of iron from Fe^2+^ to Fe^3+^ by hydrogen peroxide results in the formation of a hydroxyl free radical. This free radical is particularly dangerous, as it is non-specific and can react with most biological molecules [5]. From the labile iron pool, iron may be stored as ferritin, exported from the cell via ferroportin, or used in a variety of cellular processes (Figure 2).

**Figure 3:**
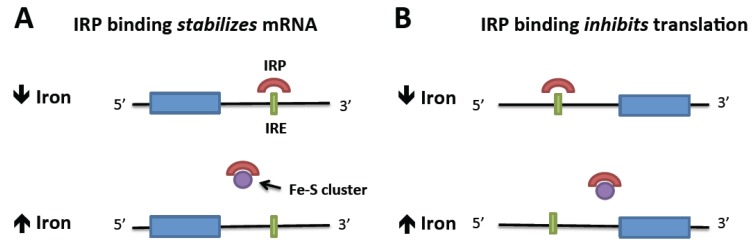
Regulation of translation by intracellular iron through IRP binding to mRNA IREs. (**A**) mRNA is stabilized by the binding of the IRP to the 3′ IRE in the absence of iron. When iron is abundant, iron-bound IRP is displaced, allowing mRNA degradation. (**B**) Translation is repressed by the binding of an IRP to a 5′ IRE. Iron abundance removes the IRP and allows translation to proceed.

A key mechanism by which cellular iron uptake is regulated is conferred by iron regulatory elements (IREs) within the mRNA of proteins involved in iron trafficking. These IREs form short hairpin loops in the 5′ or 3′ untranslated regions of relevant transcripts, and mediate post-transcriptional regulation of expression through binding of iron responsive proteins (IRPs) [6]. IREs present at the 3′ end of an mRNA transcript mediate its *stabilization* upon binding an IRP. Regulation of TfR1 translation via these interactions is illustrated in Figure 3A. In conditions of iron abundance, IRPs remain unbound, and TfR1 mRNA is degraded, resulting in less TfR1-dependent iron uptake. When iron is depleted, IRPs bind to the TfR1 3′ IRE and the transcript is stabilized, leading to additional protein translation and consequent increased TfR1-dependent iron uptake.

In contrast, the binding of IRPs to IREs residing in the 5′ region of mRNAs *inhibits* their translation (Figure 3B). Ferritin is regulated via a 5′ IRE, with the consequence that more ferritin is translated under conditions of high cellular iron levels, while ferritin production is diminished in iron-depleted cells. IREs have been identified in several other proteins involved not only in iron homeostasis, but also in cellular response to oxidative stress, cell cycle regulation, and heme synthesis [7,8].

Thus far, two IRPs––IRP1 and IRP2––have been identified and characterized [6]. These proteins have overlapping functions within the cell, but are regulated in different ways and have some distinct activities. It is likely that IRP2 is the primary regulator of cellular iron homeostasis, with IRP1 serving an accessory function [9]. In the presence of abundant cellular iron, IRP1 contains an iron–sulfur cluster that is necessary for its function as the cytosolic aconitase, catalyzing the conversion of citrate to isocitrate [10]. In iron-depleted conditions, the iron–sulfur cluster is absent and IRP1 then functions as an RNA binding protein. IRP2 lacks an Fe–S cluster and does not have a known alternate enzymatic function. It is abundant in iron-deficient cells, but is ubiquinated and targeted for proteasomal degradation in the presence of adequate cellular iron.

## 3. Iron Homeostasis in the Neoplastic Cell

It has long been recognized that there are differences in iron metabolism in the neoplastic cell compared with the normal cell. Malignant cells demonstrate a greatly increased requirement for iron as a result of their rapid cell division. The increased requirement for DNA synthesis is accompanied by increased expression of the iron-dependent enzyme ribonucleotide reductase (RR) [11]. To accommodate these augmented iron requirements, neoplastic cells increase the uptake of iron from the microenvironment. This is done primarily through the upregulation of *TFR1*, with consequent increased cell surface TfR1 expression. However, it has also been suggested that tumor cells may have alternate routes of iron uptake––these routes may be critical in achieving increased intracellular iron levels under conditions in which TfR1 is saturated. Non-receptor-mediated pinocytosis has been suggested as a significant mechanism of iron uptake in human hepatoma and melanoma cell lines after saturation of TfR1 occurs [12,13]. These alternative methods of iron uptake require further investigation.

Overexpression of the Myc protein has been identified in a wide variety of human cancers. The Myc family proteins include c-Myc, *N*-Myc, and l-Myc, all of which have been found to be upregulated in human malignancies [14]. Myc proteins are transcription factors that are involved in initiation or repression of target gene transcription by complexing with the Max protein. Myc binding sites have been identified in the regulatory regions of many genes, and Myc proteins are involved in several aspects of cellular metabolism, macromolecule biogenesis, and cell cycle regulation [15,16]. Myc proteins, specifically c-Myc, directly affect iron homeostasis and cause an increase in cellular iron availability. c-Myc overexpression increases the labile iron pool through increased expression of IRP2, and repression of ferritin heavy chain transcription [17]. This increase in available intracellular iron is directly beneficial to growth and proliferation of malignant cells.

## 4. Effects of Iron Deficiency

The effects of iron deficiency at both the cellular and organismal levels are vast and complex. As the fundamental role of iron in cellular processes (including cell cycle regulation, cellular respiration and metabolism, and cell growth and proliferation) is more clearly elucidated, the effects of iron deficiency will be better understood. Here we focus on what is known about the effects of iron deficiency at the cellular level, particularly as it pertains to proliferation and survival of the neoplastic cell.

### 4.1. Cell Cycle Regulation

Iron plays an important role in progression through the cell cycle. Importantly, iron is an essential cofactor for ribonucleotide reductase (RR), which catalyzes the rate-limiting step in DNA synthesis, the reduction of ribonucleotides to deoxyribonucleotides [11]. RR is comprised of two protein subunits. The R1 subunit is a homodimer, which is constitutively expressed in the cytoplasm and contains the catalytic and substrate-binding domains of RR. The R2 subunit is a heterodimer of RNR2 and RNR4, which migrates from the nucleus to the cytoplasm when activated. R2 contains a tyrosyl radical that is stabilized by iron and is essential for catalysis [18]. Because RR activity is required for the cell to conduct DNA synthesis and move through the S phase of the cell cycle, prolonged exposure to iron chelators results in a G1/S arrest [19].

Recently, an alternative form of the R2 subunit has been discovered that specifically functions to supply deoxyribonucleotides for DNA repair in response to DNA damage. While this alternative form is expressed at basal levels in a variety of cell types, its expression is dramatically increased by p53 and is thus called p53R2 [20]. p53R2 has also been found to contain a central tyrosyl radical stabilized by iron [21].

p53 is a critical regulator of the cell cycle which normally functions to stimulate DNA repair, cause a cell-cycle arrest at G1/S, or trigger apoptosis. It is primarily regulated by mouse double minute gene 2 (MDM2), which binds p53 and targets the protein for ubiquitination and degradation. p53 may be affected by a variety of cellular signals, including HIF-1a and ATM expression, and p53 protein levels can be increased by oxidative stress, DNA damage, abnormal cell growth, or hypoxia [22,23]. Multiple reports have shown that iron chelation increases p53 transcription, translation, and DNA binding affinity [24,25,26], resulting in G1/S cell-cycle arrest and/or apoptosis. A recent study, however, demonstrated that iron chelation with deferoxamine (DFO) causes a decrease in p53 protein due to an increase in expression of MDM2 in murine hepatocytes [27]. This may be a cell type specific effect, and further investigation into the interaction between cellular iron status and p53 expression and function is warranted.

While cellular iron levels influence p53 activity, p53 activity conversely also influences cellular iron. p53 activation has been found to reduce binding of IRP1 and IRP2 to the IREs on ferritin and *TFR1* mRNA, causing an increase in ferritin and a decrease in TfR1 expression [28]. In addition, hepcidin expression has been shown to increase in response to p53 activation [29]. p53 activation may therefore induce iron deprivation as another means of causing cell-cycle arrest.

Iron also affects one of the key downstream targets of p53, the tumor suppressor p21^CIP1/WAF1^ [30,31]. The role of p21^CIP1/WAF1^ in cell cycle regulation and cellular proliferation is complex. It acts as a cyclin-dependent kinase (cdk) inhibitor and upon activation, can lead to G1/S arrest [32]. p21^CIP1/WAF1^ achieves cell-cycle arrest primarily by binding to the cyclin E–cdk2 complex and preventing phosphorylation of the retinoblastoma protein (Rb) [33]. It also further blocks entry into the S phase of the cell cycle by directly binding to proliferating cell nuclear antigen (PCNA), the sliding clamp required for DNA polymerase to properly synthesize DNA [34]. Interestingly, low levels of expression of p21^CIP1/WAF1^ stabilize cyclin D–cdk4/6 complexes, and are therefore required for cell-cycle progression [35]. 

While the overall effect of p21^CIP1/WAF1^ on the cell cycle is a G1/S arrest, it has also been shown that degradation of p21^CIP1/WAF1^ preferentially shunts cells with DNA damage down the apoptotic pathway [36,37]. In addition, p21^CIP1/WAF1^ has been found to be elevated in several types of cancer, including breast and pancreatic cancer [38,39]. Increased p21^CIP1/WAF1^ levels in these tumors may lead to an overall anti-apoptotic effect and is suggested to be a potential mediator of chemotherapy resistance.

Iron chelation results in an overall decrease in p21^CIP1/WAF1^ protein expression, despite an increase in mRNA [24,40,41]. This is thought to occur via two primary mechanisms. First, a much lower proportion of p21^CIP1/WAF1^ transcripts translocates out of the nucleus for translation in iron-depleted cells than in iron-replete cells. Second, iron depletion induces degradation of the p21^CIP1/WAF1^ protein via the proteasome through an ubiquitin-independent mechanism. In contrast, in iron-replete cells, p21^CIP1/WAF1^ degradation occurs via an ubiquitin-dependent mechanism and at a much slower rate [42]. The reduction of p21^CIP1/WAF1^ in cancer cells by iron depletion may therefore abrogate the anti-apoptotic effects of this molecule.

Cyclins interact with cyclin-dependent kinases (cdks) to form catalytic heterodimers that directly regulate cell-cycle progression [43]. The cyclin most consistently and dramatically inhibited by iron chelation is cyclin D [44,45,46]. Cyclin D forms a complex with cdk4 or cdk6 during G1 to promote expression of the cyclin E–cdk2 complex and hyperphosphorylation of Rb [43]. Therefore, it is essential for progression into the S phase, and its degradation has been found sufficient to cause G1 arrest [47]. Iron chelation appears to cause ubiquitin-independent proteasomal degradation of Cyclin D, in a manner similar to the effect on p21^CIP1/WAF1^ [46].

Finally, iron deficiency has been found to decrease the protein expression of cyclins A, B1, D1, D2, and D3 and to increase expression of cyclin E in a neuroblastoma cell line [44]. The same study found that iron depletion decreased cdk2 expression. The cyclin E–cdk2 complex promotes passage through G1 into the S phase. With decreased cdk2 protein levels, fewer cyclin E–cdk2 complexes could form, undermining the effect of increased cyclin E expression [44]. The net effect of iron depletion on cyclin E–cdk2 activity is therefore cell-cycle arrest due to the decreased cdk2 expression.

Thus, iron plays a myriad of critical roles in progression through the cell cycle, and its deficiency can significantly inhibit cell proliferation.

### 4.2. Cellular Metabolism

Iron deficiency can profoundly affect cellular metabolism by altering the expression and/or function of proteins that are central to the regulation of cellular metabolism and cellular respiration. Several proteins involved in metabolism have been implicated in a variety of malignancies, including enzymes of the citric acid cycle, the Myc family of proteins, and the hypoxia inducible factors 1 and 2.

Iron regulates the activity of several enzymes involved in the citric acid cycle. Aconitase, citrate synthase, isocitrate dehydrogenase (IDH), and succinate dehydrogenase (SDH) are all decreased in the face of iron deprivation [48]. Aconitase and SDH both have an essential iron–sulfur cluster at their core, and their activity is therefore directly regulated by the presence or absence of iron. In contrast, citrate synthase and IDH are not regulated by the IRE/IRP system, but may be influenced by the effect of iron on the availability of their substrates. As a result of iron depletion, the net effect of changes in these enzymes’ activity is a decrease in oxidative phosphorylation, and a subsequent decrease in ATP production. Consequently, iron depletion causes a shift in cellular metabolism toward glycolysis [48]. Manipulation of the citric acid cycle is of particular interest, given recent studies that have linked mutations in several enzymes of this cycle, including IDH1/2, SDH, and fumarate hydratase (FH), with the pathogenesis of acute myeloid leukemia [48,49].

As previously mentioned, c-Myc directly affects iron homeostasis in the cell. However, cellular iron levels also affect Myc protein expression. Iron abundance has been shown to induce c-Myc overexpression in human synovial fibroblasts [50]. In addition, chelation of iron has been correlated with decreased transcription of the *MYCN* oncogene in several neuroblastoma cell lines [51]. Not only do Myc proteins participate in regulation of the cell cycle as outlined above, they are also involved in the regulation of basic cellular metabolism. Lactate dehydrogenase A (LDH-A) has been identified as a target gene transactivated by c-Myc [52]. LDH-A catalyzes the final step of glycolysis, converting pyruvate to lactate. Hence, altered iron levels could affect glucose metabolism in neoplastic cells through modulation of Myc protein levels.

Hypoxia-inducible factor 1 (HIF1) is a heterodimeric transcription factor composed of alpha and beta subunits; upon dimerization, HIF1 translocates to the nucleus and regulates gene expression [53]. The beta subunit of HIF1 is constitutively expressed. The activity of the HIF1 heterodimer is therefore primarily regulated by the amount of the alpha subunit available for dimerization. HIF1 binds to hypoxia response elements (HREs) on target genes to initiate transcription. The targets of HIF1 can be grouped loosely into four categories; angiogenic factors, survival factors, invasion/metastasis factors, and glucose transporters/glycolytic enzymes. The net effect of HIF1α stabilization is generally thought to be cell survival, proliferation, and in the case of malignant cells, metastatic invasion. HIF1α upregulation has been reported in a number of human malignancies, including breast, lung and colon carcinomas [54]. In many cases, high levels of HIF1α are associated with poorer overall prognosis. However, some studies have suggested that increased HIF1α expression correlates with improved survival in renal cell carcinomas and neuroblastomas [55,56]. The role of HIF1α in maintenance and progression of malignant disease is complex, and likely tumor type specific.

HIF1α is normally negatively regulated by the action of HIF prolyl hydroxylases, which hydroxylate the oxygen dependent domain of HIF. This targets the protein for degradation by the von Hippel Lindau (VHL) protein, which is an E3 ubiquitin ligase. Hypoxia is the primary mechanism that leads to HIF1α stabilization; however, other signals, such as the IGF-1 and VEGF growth factors, have also been found to stimulate the hypoxic response. Iron deficiency activates the hypoxia response under conditions of normoxia, which ultimately results in stabilization of HIF1α [57]. The impact of this in the setting of malignancy is unclear, given the complex transcriptional activity of the HIFα proteins in individual tumor types.

### 4.3. Metastatic Potential

*N*-Myc downstream regulated gene-1 (NDRG-1) has been associated with reduced metastatic potential in several malignancies. NDRG-1 is a ubiquitously expressed protein whose function has not been well elucidated, but is likely tissue specific [58]. In general, NDRG-1 expression is associated with decreased proliferation and increased cellular differentiation. Its transcription is induced by cellular stress signals, and has been found to be regulated by a number of upstream factors, including the Myc family proteins, HIF1α, PTEN, and p53 [58]. Iron chelation also results in upregulation of NDRG-1, via a mechanism that does not appear to involve IRP/IRE, and is independent of both p53 and HIF1α [59]. NDRG1 has been examined in several human malignancies because of its association with decreased cellular proliferation, and in most instances, it is regarded as a metastasis repressor [60,61]. It is worth noting, however, that there are some malignancies, such as hepatocellular carcinoma, in which NDRG-1 has been associated with more aggressive disease and an increased metastatic burden [62,63].

## 5. Iron Chelators

Iron chelation has been explored as an adjunct to therapy, or in some cases as monotherapy, in the treatment of a wide variety of human cancers, as will be examined in detail below. As long ago as the early 1980s, the anti-proliferative effects of iron chelation were being explored in leukemias, neuroblastoma, and breast cancer [64,65,66]. Iron chelation may be effective as an anti-neoplastic agent via two distinct mechanisms. The first is through iron depletion. Limiting cellular iron availability may interfere with a number of cellular processes that are vital to cell proliferation, as outlined above. The second potential mechanism of action is via formation of a complex that increases iron’s redox potential, as described in further detail below. This redox cycling of iron produces toxic free radicals within the neoplastic cell, ultimately leading to cellular damage and death.

**Figure 4:**
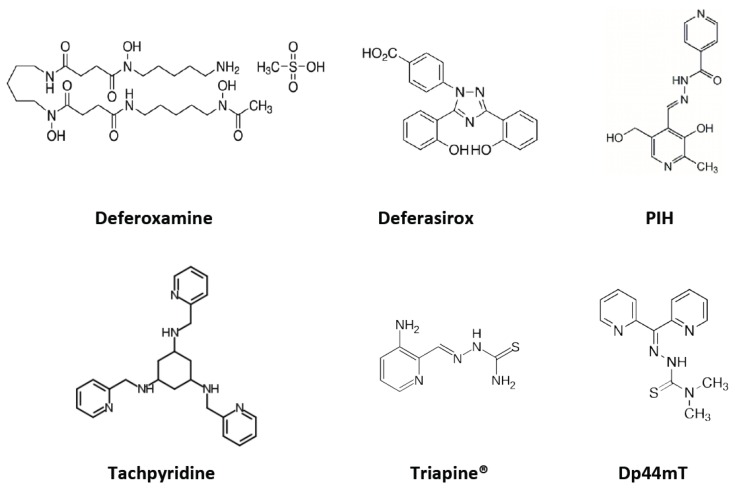
The chemical structure of several key iron chelators. Abbreviations: PIH––pyridoxal isonicotinoyl hydrazone; Dp44mT––di-2-pyridylketone-4,4,-dimethyl-3-thiosemicarbazone.

A number of different classes of iron chelators have been used as anti-cancer therapies, in both *in vitro* and *in vivo* studies (Figure 4). These chelators differ in structure, function, and anti-proliferative activity. Certain structural elements significantly impact iron chelator potency. The first is lipophilicity; more hydrophobic compounds are better able to cross the cell membrane and chelate the pool of intracellular iron. In addition, lipophilic chelators that gain access to the intracellular space may also directly bind the iron at the core of RR, improving anti-proliferative efficiency [67]. The second relevant structural property of an iron chelator is its ability to allow bound iron to participate in redox reactions within the cell. Hexadentate chelators fully occupy iron’s coordination sphere, rendering it unable to participate in cellular redox reactions. In contrast, bidentate or tridentate chelators are capable of partially dissociating with iron, thus allowing it to participate in chemical reactions. Finally, chelators that possess nitrogen as a donor atom are able to bind both ferric and ferrous iron with almost equal affinity, thus allowing the iron atom to be reduced or oxidized [68]. As mentioned previously, the redox potential of iron generates cytotoxic reactive oxygen species, an advantage when the goal of chelation is an anti-neoplastic effect.

The major classes of iron chelators that have been used as anti-neoplastic agents will be reviewed here, followed by a discussion of tumor-specific anti-proliferative effects of iron chelation.

### 5.1. Deferoxamine

Deferoxamine (DFO) was the first of the iron chelators to be examined for its anti-neoplastic effect. It is a siderophore that has historically been used for the treatment of iron overload disorders, such as thalassemia and transfusional iron overload. DFO is poorly lipophilic and does not enter the cell via passive diffusion. Therefore, it primarily causes a reduction in total body iron by chelating extracellular iron. DFO has a selective high affinity for Fe^3+^ and binds iron in a hexadentate fashion, thus rendering the atom unavailable to participate in redox chemistry and free radical formation [69]. While DFO is commonly used in patients, its poor membrane permeability and inability to permit redox cycling of iron are disadvantageous when the goal is an anti-proliferative effect. In addition, DFO is poorly absorbed from the intestine, must be given by intravenous route, and has a very short plasma half-life. These disadvantages led to the search for more effective chelators that are easier to administer to patients.

### 5.2. Deferasirox

Deferasirox (Exjade™; DFX) is a synthetic tridentate iron chelator that is very efficient and highly selective. It is highly lipophilic, rendering it membrane permeable. It also allows iron to participate in redox chemistry and generate reactive oxygen species [70]. DFX is orally bioavailable and currently being used clinically for the treatment of iron excess arising from transfusion overload. Chelation with DFX results in a G1/S arrest and increased apoptosis in treated cell lines [71]. Studies of the use of this compound as an anti-neoplastic agent are very preliminary; however, there is some suggestion that DFX may have an anti-proliferative effect in myeloid leukemia and hepatoma cell lines [72,73].

### 5.3. Tachpyridine

Tachpyridine (Tachpyr) is a synthetic hexadentate divalent metal chelator. It has been shown to bind multiple metals, including copper and zinc, but its cytotoxic effects are likely mediated by iron deprivation, as iron replacement abolishes these effects *in vitro* [74]. Tachpyridine’s cytotoxicity is initially mediated by iron depletion, but after prolonged *in vitro* application, Fe^2+^ complexed with tachpyridine will also participate in redox chemistry [75]. More recent study of this compound has shown that it is capable of inducing a G2 cell-cycle arrest, and it may act as a radiosensitizer in human colon carcinoma cell lines [76]. Cellular iron depletion typically results in a G1 arrest, as outlined above. It is unclear how to reconcile this fact with the G2 arrest seen with tachpyridine use, although the compound’s ability to also chelate zinc and copper may play a role in explaining this discrepancy.

### 5.4. Aroylhydrazones: Pyridoxal Isonicotinoyl Hydrazone (PIH) Analogs

Pyridoxal isonicotinoyl hydrazone (PIH) analogs comprise a class of lipophilic tridentate iron chelators that readily cross the plasma membrane [77]. This feature may be partly responsible for the very efficient iron chelation exhibited by these compounds. PIH analogs bind iron in a specific manner, with very little affinity for other chelatable metals such as copper or manganese [78]. An additional advantage of this class of iron chelators is their oral bioavailability. Studies in animal models of iron overload have found that orally administered PIH is effective at increasing daily iron excretion and decreasing splenic and hepatic iron content [79,80]. Subsequent generations of aroylhydrazones have been synthesized with increasing ability to chelate iron and to inhibit cell proliferation. The most recent series of compounds are the di-2-pyridylketone isonicotinoyl hydrazone (PKIH) analogs, which have shown the greatest anti-proliferative effect in this class of chelators [81].

### 5.5. Thiosemicarbazones

The thiosemicarbazones comprise a class of synthetic tridentate metal chelators that not only bind iron, but will also chelate other metals including copper and zinc. Because of their tridentate structure, thiosemicarbazones allow iron to participate in redox reactions. Drugs in this class have been shown to be potent inhibitors of RR. Some of these compounds have specifically been shown to bind iron at the active site of the enzyme and, in an oxygen dependent fashion, destroy the tyrosyl radical that is critical for enzyme function [82]. Early thiosemicarbazones were subject to glucuronidation and rapid excretion from human subjects in a phase I trial of 5-hydroxy-2-formylpyridine thiosemicarbazone (5-HP) [83]. Newer drugs in this class, for example 3-aminopyridine-2-carboxaldehyde (Triapine^®^), are resistant to glucuronidation and are therefore not excreted as rapidly [84]. Triapine^®^ has been used as an anti-neoplastic agent in phase I and II trials, as detailed below.

### 5.6. di-2-Pyridylketone Thiosemicarbazone (DpT) Series

The di-2-pyridylketone thiosemicarbazones comprise a class of synthetic “hybrid” iron chelators. The di-2-pyridylketone isonicotinoyl (PKIH) analog aroylhydrazones were found to possess greater anti-proliferative effects when compared with earlier compounds of that class [81]. The di-2-pyridal backbone from these compounds was then combined with the thiosemicarbazide moiety found to mediate the anti-proliferative activity of the thiosemicarbazone class of chelators [85]. This series of chelators has demonstrated high affinity binding to Fe^2+^, the ability to allow bound iron to participate in redox reactions and free radical formation, and potent anti-proliferative effect against several neoplastic cell lines *in vitro* [85]. The most potent of these chelators, di-2-pyridylketone-4,4,-dimethyl-3-thiosemicarbazone (Dp44mT), has *in vivo* efficacy in a mouse model of human tumor xenografts [86].

### 5.7. Other Iron Chelators

Several pharmaceutical agents, in clinical use for other indications, have been serendipitously discovered to also function as iron chelators. Ciclopirox olamine, a compound developed for use as an antifungal agent, was identified during a large chemical library screen to have an anti-leukemic effect [87]. The investigators determined that this effect was the result of iron chelation.

While studying the effect of eltrombopag on megakaryopoiesis in patients with myelodysplastic syndrome, it was discovered that this compound had an inhibitory effect on proliferation of blasts in this setting [87]. Further investigation confirmed eltrombopag inhibited the proliferation and induced the differentiation of myeloid leukemia cells, and identified iron depletion as the mechanism behind these effects [88].

Finally, a relationship between iron depletion and Wnt signaling has recently been elucidated. Aberrant activation of the canonical Wnt/β-catenin signaling pathway has been found in multiple human neoplasias [89]. Wnt activation causes stabilization of the β-catenin protein, which can translocate to the nucleus and activate the transcription of Wnt target genes such as c-Myc that mediate cell proliferation. Epidemiological studies have implicated dietary iron in the pathogenesis of colorectal cancers for which activating mutations of the Wnt pathway play a crucial role [90,91]. Moreover, iron has been shown to upregulate Wnt/β-catenin signaling and stimulate cellular proliferation in colorectal cell lines harboring Wnt activating mutations [92]. The role of iron in Wnt signaling has since been corroborated by studies in which iron chelation was fortuitously shown to antagonize β-catenin. Two independent groups conducting high throughput screens designed to identify novel Wnt/β-catenin inhibitors discovered molecules with iron chelating properties as their top hits [93,94]. Song and colleagues identified a group of acyl hydrazones capable of destabilizing β-catenin. They showed that excess iron neutralized this property. Furthermore, treatment with structurally unrelated iron chelators, such as deferasirox, similarly inhibited activation of Wnt targets and the growth of colorectal cancer cells with aberrant activation of Wnt/β-catenin. Coombs *et al.* discovered that the molecule HQBA, identified for its Wnt inhibitory properties, is a potent chelator of Fe^2+^. They confirmed that iron chelation mediates its anti-Wnt activity and demonstrated its ability to delay the development of murine mammary tumors. Iron chelation was also shown to inhibit Wnt/β-catenin in human subjects; a decrease in expression of the Wnt/β-catenin target gene AXIN2 was seen in a small cohort of leukemia patients treated with ciclopirox [93]. These results warrant further studies to determine the extent to which the anti-tumor effect of iron chelation is mediated by an inhibition of the Wnt/β-catenin pathway.

### 5.8. Other Targeted Therapies

A number of additional approaches have been taken to target cancer cells through proteins associated with iron metabolism. The best studied of these proteins is the transferrin receptor. Because it has been shown that many types of cancer cells exhibit increased surface levels of the transferrin receptor (TfR), attempts have been made to various cytotoxic agents to transferrin, with the goal of targeting that cytotoxic agent to cancer cells through endocytic uptake by the TfR (reviewed in [95]). Other iron-associated proteins being investigated with the intent of targeting human cancers include the 6-transmembrane epithelial antigen of prostate (STEAP) family of proteins. These proteins largely function as metalloreductases, aiding in cellular iron import [96]. Some of these STEAP proteins have been found to be overexpressed in a variety of cancers, including prostate, colon, and breast, leading to their identification as potential immunotherapeutic targets [97,98]. Details of these targeted approaches are beyond the scope of this review.

## 6. Iron Chelation as a Treatment for Cancer

Iron chelation as a therapeutic intervention has been examined both *in vitro* and *in vivo* in a variety of human malignancies. The most studied applications of iron chelation as an adjunct to therapy have been in hematopoietic malignancies and neuroblastoma. The role of chelation has also been examined in several other solid tumors, including melanoma, breast cancer, and epithelial carcinomas, such as those of the prostate, esophagus, and colon.

### 6.1. Leukemia

#### 6.1.1. *In Vitro* Studies

The use of iron chelation as a possible adjunct to therapy has been most extensively studied in acute leukemias. Treatment of acute myeloid leukemia (AML) cell lines with DFO or DFX reproducibly shows an anti-proliferative effect, which is irreversible after prolonged (48–72 h) exposure. An increase in apoptosis has also been demonstrated in AML cell lines under these conditions. These effects are less pronounced in non-malignant control cell lines [72,99]. Newer iron chelators have also been tested against leukemias. The synthetic chelator Dp44mT has demonstrated *in vitro* efficacy against an acute promyelocytic leukemia cell line. This was accompanied by caspase-3-mediated induction of apoptosis and decreased proliferation with a G1/S arrest. These effects were far greater than what was measured in control non-proliferating cells [100]. Ciclopirox is an anti-fungal agent that was serendipitously found to act as an iron chelator. It has been shown *in vitro* to have preferential cytotoxicity toward acute leukemia and multiple myeloma cell lines when compared with non-malignant controls, although the therapeutic window observed was small. *In vitro* synergism of cytarabine and ciclopirox was also observed in these leukemia lines [87]. Eltrombopag––a thrombopoiesis stimulating agent that inhibits proliferation of leukemia cells––was surprisingly found to function as an iron chelator, and caused a G1/S arrest and cytotoxicity in both human and murine leukemia cell lines [88].

Not only has iron chelation been used to decrease cell proliferation and induce apoptosis in acute leukemias, but it has also been demonstrated to induce monocytic differentiation *in vitro*. Callens *et al.* demonstrated that both DFO and DFX, in conjunction with vitamin D3, were able to induce monocytic differentiation in AML cell lines [101]. This effect was found to be dependent on activation of the MAPK pathway, including ERK, JNK, and p38 MAPK [101]. Eltrombopag was also found to induce differentiation in murine and human AML cell lines, in a manner dependent on iron depletion [88].

Depriving leukemia cells of iron has also been achieved by targeting the transferrin receptor with antibodies. Ch128.1 is a mouse/human chimeric anti-TfR1 antibody which has been used to take advantage of the increased surface TfR1 found on malignant B cells to target this population. This antibody does not block binding of Tf to TfR but it reduces their internalization, resulting in cellular iron deprivation. While there was some variability in response, *in vitro* use of ch128.1 did cause p53-dependent cytotoxicity in a subset of malignant hematopoietic cells [102].

#### 6.1.2. *In Vivo* Studies

Multiple groups have described the ability of iron chelators to decrease leukemic tumor growth in mouse xenograft models of AML. Decreased tumor burden was observed in xenograft models of AML treated with intraperitoneal DFO [101], or intratumoral DFX in a model of subcutaneous injection of leukemia cells [72]. In addition, decreased engraftment with DFX treatment was seen in murine model of AML, suggesting a possible specific effect of iron chelation on the leukemia-initiating cell, although this area requires further investigation [72]. Finally, when eltrombopag was examined in a mouse model of AML, increased differentiation of the leukemic blast population was observed, mirroring what had been seen *in vitro*. In addition, there was prolonged survival in eltrombopag treated animals, compared with controls [88].

Because of these encouraging preclinical results, iron chelation has been tried in human subjects with relapsed or refractory acute leukemias. These studies are largely case reports and small case series, but show some encouraging results nonetheless. A 73-year-old man with relapsed, refractory AML receiving palliative supportive care with repeated blood transfusion, who developed iron overload and was treated with DFX was found to be in cytogenetic complete remission from his leukemia after 12 months of chelation with no additional chemotherapy [103]. A 69-year-old man with relapsed AML was treated with DFO and vitamin D as differentiating agents, without additional chemotherapy. He had a decrease in peripheral blast count, decreased requirement for transfusion support, and increased monocytic differentiation [101].

Iron chelation has also been tried in combination with traditional chemotherapy. Estrov *et al.* report the case of a six-week-old infant with pre-B ALL, who failed induction chemotherapy [104]. Re-induction was undertaken with DFO followed by DFO and cytarabine. An *in vitro* blast colony assay performed after DFO monotherapy revealed a decreased number of blasts and increased blast differentiation. A bone marrow evaluation at the end of a 20-day treatment course showed monocytic differentiation. Stabilization of peripheral blood blast count was seen after 48 h of treatment and the peripheral blast count decreased to zero after 15 days of combination therapy was initiated [104].

Finally, a phase I study of Triapine^®^ and cytarabine was undertaken for adults with AML or high-risk myelodysplastic syndrome. There were 31 evaluable patients. Four patients demonstrated a complete response, with the remaining 27 individuals having no response. The median survival for responders was 30.9 weeks *vs.* 12.6 weeks for all patients [105]. For many of these reports, there is little to no long term follow-up, and so the duration of effect is unclear. Despite this, these early results merit further investigation.

### 6.2. Lymphoma

While many of the studies in hematopoietic malignancies have been performed on acute leukemias, use of iron chelation has also been examined as an adjunct to therapy in lymphomas and shown encouraging *in vitro* results. Choi *et al.* treated multiple human lymphoma cell lines with DFX and found cytotoxicity accompanied by caspase-3/caspase-9-mediated apoptosis [106].

Mantle cell lymphoma is a type of non-Hodgkin lymphoma characterized by the t(11;14) translocation, and is associated with increased cyclin D1 expression. Mantle cell lymphoma is typically aggressive and only hematopoietic stem cell transplantation is curative. Following treatment with DFX, human t(11;14) cell lines showed decreased expression of cyclin D1, and increased caspase-3-mediated apoptosis [107]. These are encouraging preliminary preclinical data for a tumor that could benefit from novel therapeutic options.

### 6.3. Neuroblastoma

Iron chelation has also been studied in a number of non-hematologic malignancies. Neuroblastoma is the second most commonly detected solid tumor in pediatric patients [108]. Prognosis is poor (<40% survival) in the face of advanced stage disease or certain biological features, such as *MYCN* amplification. As such, novel therapeutic approaches continue to be explored. Neuroblastoma has been shown to be relatively sensitive to the effects of iron chelation *in vitro*. DFO has been the most frequently studied chelator, and multiple groups have found an anti-proliferative effect of this compound when applied to neuroblastoma cell lines, with relatively little effect on non-malignant controls [109]. Others have further demonstrated an increase in apoptosis in neuroblastoma cell lines compared with non-neuroblastoma solid tumor lines when treated with iron chelators. In addition, iron chelation with DFO mediated a decrease in *N*-Myc expression in *MYCN* amplified neuroblastoma cell lines, likely at a transcriptional level. This is intriguing given the importance of *MYCN* amplification as a marker of poor prognosis in neuroblastoma [51]. Some of the newer synthetic iron chelators have also been studied in neuroblastomas. The PIH analog 311 and Triapine^®^ both demonstrated a greater anti-proliferative effect on neuroblastoma cell lines in comparison with DFO. Again, relatively few negative effects were seen in several tested normal cells, including fibroblasts, and bone marrow progenitor cells [110].

With these promising preclinical results, iron chelation has been used in small case series as either monotherapy or as part of combination chemotherapy in patients with neuroblastoma. One trial used a single eight-day course of DFO at 150 mg/kg/day as monotherapy in nine neuroblastoma patients [111]. Six of the nine patients had received previous treatment with traditional chemotherapy. Seven of these patients showed a greater than 50% reduction in bone marrow involvement and one patient’s bone marrow was completely cleared of neuroblastoma cells (although that child’s initial level of marrow infiltration was only 2%). There were six patients with measurable tumor at start of therapy and one of these had a nearly 50% decrease in tumor volume, with the others maintaining stable tumor size. A second clinical trial using DFO as monotherapy in children with relapsed neuroblastoma used two different dosing regimens. At both dosing levels, the patients were treated five days/week, every other week. The lower dose used was 120 mg/kg/day, and no partial or complete responses were seen. The higher dose was 240 mg/kg/day, and all patients treated with this experienced unacceptable toxicities [109]. The European D-CECaT trial, which used DFO in combination with cyclophosphamide, etoposide, carboplatin, and thiotepa showed some promising preliminary results. This was a single arm trial for advanced stage neuroblastoma, and included 57 evaluable patients. Post-surgical responses included 24 complete responses, 26 partial responses, 3 minor responses, and 4 patients with progressive disease [112]. Similar to the small trials/case series in the leukemia population, these early promising results merit further investigation.

### 6.4. Solid Tumors Other than Neuroblastoma

Although the hematologic malignancies and neuroblastoma comprise the majority of tumor types that have been studied, iron chelators have been examined in other tumors. DFX is an appealing agent as it is a very effective iron chelator and can be orally administered. It has been tested *in vitro* against lung carcinoma, neuroepithelioma, and esophageal carcinoma, and has been found to inhibit proliferation in all of these tumor types [113,114]. DFX has also been used in xenograft mouse models of lung cancer, and been found to inhibit tumor growth with essentially no toxic effect to the normal tissue [113]. Dp44mT is a very efficient synthetic iron chelator with marked anti-proliferative effects on hematopoietic malignancies. It has been tested in prostate cancer cell lines, where it was found to have an anti-tumor effect mediated by NDRG1 [115]. Dp44mT was also found to have anti-proliferative activity in breast cancer cell lines, both when used alone or as an adjunct to doxorubicin [116].

## 7. Conclusions

Iron plays key roles in cellular metabolism, growth and proliferation. Iron depletion causes a shift in cellular metabolism from oxidative phosphorylation to glycolysis, reducing the total number of ATP molecules available for cellular processes. The downstream metabolic effects of iron depletion on Myc-mediated transcription and the hypoxic response are complex and still being elucidated. Iron depletion also causes cell-cycle arrest and inhibits cellular proliferation through the inhibition of RR, induction of p53, and degradation of cyclin D1. Finally, iron chelation has been shown to increase degradation of p21, resulting in an increase in apoptosis.

Because iron depletion has been shown to have an anti-proliferative effect, there has been a great deal of interest in using iron chelation in the treatment of cancer. A number of iron chelators have been developed and tested with the goal of using iron chelation as an adjunctive therapy. *In vitro* results have been promising, with anti-proliferative and differentiating effects seen in myeloid leukemias, and anti-proliferative effects in non-Hodgkin lymphoma. *In vitro* assays have also been encouraging in solid tumors, including neuroblastoma, prostate cancer, breast cancer, and colon carcinoma.

The effects of iron chelation on these malignancies *in vivo* have been somewhat more variable. This may reflect the remarkable ability of the organism as a whole to maintain homeostasis in the face of iron depletion, the ability of the tumor to adapt to changing circumstances *in vivo*, or the complex interplay of iron homeostasis with cellular growth, metabolism, and proliferation. Further study is therefore required to clarify the potential applications of iron chelation as an adjunct to therapy in the treatment of neoplastic disease.
